# Efficient technique of microarray missing data imputation using clustering and weighted nearest neighbour

**DOI:** 10.1038/s41598-021-03438-x

**Published:** 2021-12-21

**Authors:** Aditya Dubey, Akhtar Rasool

**Affiliations:** Department of Computer Science & Engineering, Maulana Azad National Institute of Technology, Bhopal, 462003 India

**Keywords:** Computational biology and bioinformatics, Genetics

## Abstract

For most bioinformatics statistical methods, particularly for gene expression data classification, prognosis, and prediction, a complete dataset is required. The gene sample value can be missing due to hardware failure, software failure, or manual mistakes. The missing data in gene expression research dramatically affects the analysis of the collected data. Consequently, this has become a critical problem that requires an efficient imputation algorithm to resolve the issue. This paper proposed a technique considering the local similarity structure that predicts the missing data using clustering and top K nearest neighbor approaches for imputing the missing value. A similarity-based spectral clustering approach is used that is combined with the K-means. The spectral clustering parameters, cluster size, and weighting factors are optimized, and after that, missing values are predicted. For imputing each cluster’s missing value, the top K nearest neighbor approach utilizes the concept of weighted distance. The evaluation is carried out on numerous datasets from a variety of biological areas, with experimentally inserted missing values varying from 5 to 25%. Experimental results prove that the proposed imputation technique makes accurate predictions as compared to other imputation procedures. In this paper, for performing the imputation experiments, microarray gene expression datasets consisting of information of different cancers and tumors are considered. The main contribution of this research states that local similarity-based techniques can be used for imputation even when the dataset has varying dimensionality and characteristics.

## Introduction

In the last few years, since the big data age started, the sophisticated data collection tools and the amount of data generated have reached an unprecedented example^1^. Many methods can be implemented to collect observational details, such as automated and continuous sensor monitoring or repeated findings (surveys, medical reports, etc.)^2^. DNA microarray has become an extremely effective research instrument with the advancement in biotechnology so that organism’s complexities in genetics can be identified^3^. By evaluating these datasets, relevant information and overviews may be obtained. Biological and scientific research utilize datasets of this application. Some examples of research include identifying gene function, analyzing gene regulation network^4^, detection of new disease-related pathogens^5^, and drug effect recognition^6^. For this, researchers implement complex model-based machine learning and predictive analysis methods to evaluate microarray data for identifying relevant biological information.

Preprocessing is a stage or phase where data may be detected, sorted, or dealt with several anomalies. Unfortunately, due to rough working conditions or unregulated causes, including adverse weather, system malfunction, or unreliable signal, the gene expression data generated usually contain missing values^7^. One typical example of missing data is a weak spot; another example of missing value is technical faults when hybridizing; the third example of missingness includes dust, marks, and errors in the slides. The fourth example is inaccurate resolution corrupted image. Studies have shown that specific, easily accessible microarray datasets contain missing data entries ranging from 50 to 95%. Due to the availability of missing data, the various data mining operations such as clustering, classification, and identification of differential expressions are decremented.

The missing gene expression data give rise to three forms of concerns: first is the performance loss, second is difficulties in analyzing data, and third biased results are produced because of discrepancies between missing and available values. Although a few research methods may work with the missing values, most of the other methods need the dataset to be complete. Statistical analytical tools either handle the entire dataset or use alternative methods to fill out the data which are not available. Most data mining techniques used for the simulation arbitrarily chose value, for example, − 1, 1, 0, or 9, to make the missing value, leading to the inconsistencies in the results generated. Therefore, imputation plays a critical function in such circumstances.

Missing value imputation procedures are classified into two types: Single imputation and multiple imputation. Single imputation procedures provide only a single value to the missing instance value. Mean substitution, deck procedures, regression procedures, expectation-maximization, pairwise deletion, list-wise deletion, Naïve Bayes are some of the single imputation procedures. Compared to other data imputation techniques, multiple imputation has many merits. Multiple imputation consists of a three-step procedure namely imputation, analysis, and pooling. The principle of multiple imputation is to fill the missing values many times, resulting in many complete datasets. MICE also referred to as fully conditional specification or sequential regression multiple imputation^8^. MICE requires the implementation of a set of regression models on each variable containing missing entries to be modelled with the remaining variables of the dataset. For example, logistic regression is required for modelling the binary variables; similarly, linear regression is required for continuous variables. MICE is an iterative approach, where the loop terminates at the condition when the distribution of parameters where the number of iterations can be specified by the researchers. The decision of the number of cycles is a major issue in MICE. Secondly, the creation of the number of imputed datasets should be decided per the dataset, otherwise, the the entire imputation process becomes cumbersome. The third issue is the decision on which variables to be included in the imputation procedure. Describing bounds and restrictions is also an issue. The unavailability of theoretical justification of MICE is one of the drawbacks. Implementation of MICE required missing data to be of the type MAR, otherwise biased results are generated.

The remainder of the paper is summarized as follows: "Problem formulation"section formulates and describes the missing data challenge. "Literature review"section discusses the literature survey of research area by considering the popular approaches. The theoretical context for understanding the proposed method is described in "Related work"section. "Proposed work"section contains the implementation work of the proposed technique. In "Experiments and analysis"section, the results proves that the proposed technique makes accurate imputation as compared to other local imputation techniques. The conclusion and future work are discussed in the last section. The contributions of this paper are described as follows:

**Figure 1:**
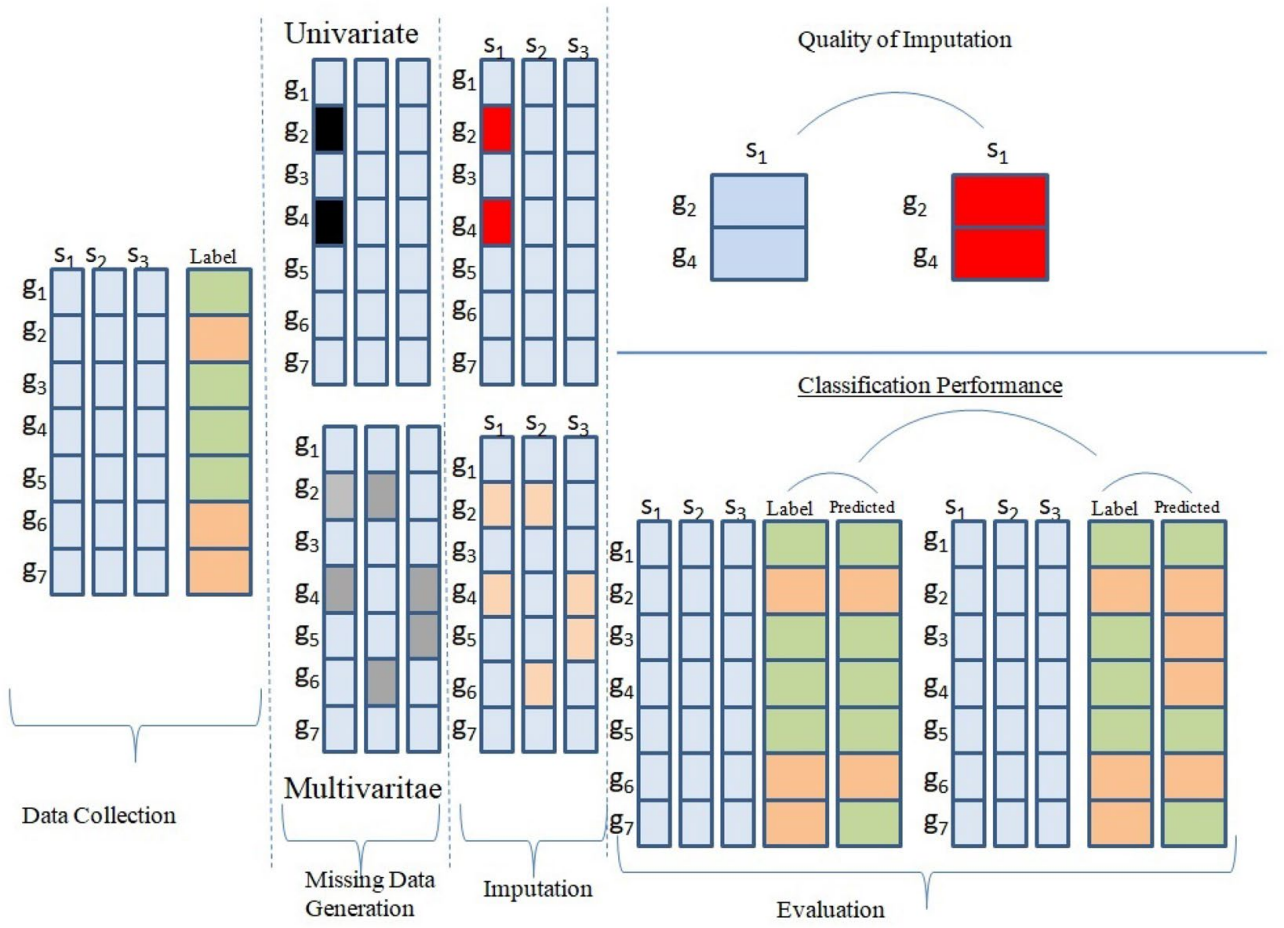
Missing value imputation procedure.

- Considering the local data similarities, a local similarity-based imputation technique is proposed that utilizes the concept of clustering algorithm for imputing the missing values. In other words, data is clustered at the initial step, followed by the weighted imputation form the nearest neighbors falling in the same cluster.
- The top KNN approach is used together with a weighted function which enhances the imputation accuracy.
- The proposed technique is implemented on four genes expressed datasets. For analyzing the performance of the proposed technique MATLAB platform is utilized. For comparing the proposed technique’s performance, five popular local imputation techniques, including KNNImpute, SKNNImpute, IKNNImpute, LLSImpute, and SLLSImpute, are used.

## Problem formulation

This paper aims to design an imputation procedure making accurate imputation of missing values. For exemplifying this problem more precisely, Fig. 1 depicts a gene expression dataset that does not contain any missing values at the initial stage. Some of the values are artificially deleted in some of the attributes $s_1, s_2,..., s_M$. Let $G= \{g_1, g_2, g_3,..., g_N\}$ represents the data collected from N different genes and $j$ th sample from $i$ th gene can be denoted as $G_{ij}$ for i={1,2,3,...,N} and j={1,2,3,...,M}. The univariate missing structure represents that missing values are available in only one of the samples whereas the multivariate missing structure represents many samples containing missing values. At the next step, imputation represents the prediction of missing values using some technique. In this paper, for comparing the performance of an imputation, Root Mean Square Error (RMSE) is used. RMSE describes the difference between the actual and predicted values. Table 1 lists the symbols and their descriptions that are used throughout this paper. In this paper, the missing data is represented by ‘?’. Additionally, for identifying whether all values of a gene are available or missing, a matrix $H\in \mathbb {R}^{N\times M}$is used.

1 $$\begin{aligned} {h_{ij}}= {\left\{ \begin{array}{ll} 1, \hbox {if value of } \hbox {G}_{\mathrm{ij}} \hbox { is present} \\ o, \hbox {otherwise} \end{array}\right. } \end{aligned}$$

here i={1,2,3,...,N} and j={1,2,3,...,M}. The Problem created by missing data in genome data is formulated as follows:

### Problem 1

 Imputation in a multivariable gene expressed dataset.

Given: a multivariable gene dataset $G\in \mathbb {R}^{N\times M}$; a boolean matrix H;

Imputation: The imputed values of the missing gene sample indicated by H.

**Table 1:** Symbols and definitions.

Symbols	Definition and description
G	Initial dataset
N	Number of data instances
M	Number of attributes
$S \in G^{N\times N}$	Similarity matrix for the N data instances $g_1,\ldots ,g_N$
$S_{ij}$	Similarity between data instance $g_i$ and $g_j$
K	Desired number of clusters
D	Degree matrix
W	Adjacency matrix
L	Graph laplacian
$c_i$	*i*th cluster
C	Set of clusters
$e_i$	*i*th eigen vector

## Literature review

Techniques of missing gene expression data are classified into four major types: global techniques, local techniques, hybrid techniques, and knowledge-assisted techniques^9^. Global techniques consider the global correlation structure of the dataset for making an imputation. Examples of global techniques include BPCA and SVD approaches. The main advantage of BPCA is that it utilizes the data from those genes which contain missing values at some of the samples^10^. This is achieved by partially using the sequential imputed value for the next level of imputation. In BPCA, selecting exact parameters is not a constraint. A procedure for the automatic selection of parameters is utilized. The approach does not fail even for the large dataset. Row or column level normalizing may lead to an inaccurate prediction. The SVD algorithm goal is to predict the missing data as a linear combination of the k-most significant eigengenes^11^. An ideal linear combination is established by regressing the incomplete variable against the k-most notable eigengenes.SVD performs well for the time series dataset, which contains low noise^11^. Global techniques’ usage becomes cumbersome when a small percentage of data is missing for a large dataset. For making an imputation, a large dataset is involved in the computation.

Unlike global techniques, local techniques aim to find a similar local structure in the dataset; here, imputation is done by those genes that have higher similarity to the target gene. KNNImpute is the first approach that imputes the target gene’s missing value with its K number of nearest neighbors^11^. KNNImpute approach does not require exact parameter selection as needed for the SVD. The Local Least Square imputation (LLSImpute) approach operates in two steps: the choice of K number of similar genes by implementing the L2 norm or Pearson coefficient. After that, the missing gene is considered to have a linear correlation with the available genes^12^. Sequential Local Least Square imputation (SLLSImpute) process uses those genes in which some sample values are present^13^. In this approach, the value of K that is the number of similar genes is not fixed and may vary as per the missing percentage. The Iterated Local Least Square imputation (ILLSImpute) approach in which the number of relevant genes utilized for the imputation is not fixed^14^. In this approach, the imputed value from the first iteration can be used for the next iteration. The approach utilizes the Local Least Square approach at each iteration. The clustering technique combined with the top KNN technique is proposed for making the imputation^15^. The method performs better for the dataset of the type temporal and spatial characteristics. Two regularized local learning techniques have been used for predicting the missing data on the gene expression dataset^16^. The training of RLLSimpute L2 regularized local least square is made on the target missing gene instance and its neighboring instances so that the imputation can be made.

In a hybrid approach Recursive Mutation Imputation, global approach(BPCA), and local approach(LLS) are combined for making imputation^17^. Missing values in the target gene decide the order of the imputation process. The support vector regression technique was also utilized for predicting the missing data in a DNA microarray gene expression using an orthogonal coding scheme^18^. For some application datasets, support vector regression performs well as compared to other imputation procedures such as KNN and BPCA. One more advantage of support vector regression is the requirement of less time for calculation. The weighted KNN combined with neural network concepts had been proposed for making imputation^7^. The auto-associative neural network is having one hidden layer consisting of input and output neurons. The interdependence of attributes can increase prediction accuracy. Fuzzy clustering technique combined with soft computing concepts is used for making imputation^19^. The technique is dependent on the optimal parameter selection. The hybrid approach called HPM-MI utilized K-means clustering together with multilayer perceptron^20^.

Some imputation techniques consider the domain knowledge of the dataset. For example, the information on the microarray dataset’s biological functionality can be regarded as in the imputation procedures. The advantage of knowledge assisted technique is that imputation procedures still perform better even if the dataset contains some noisy data. The information of Histone acetylation is used for designing an imputation procedure^21^. This knowledge is used together with LLSImpute and KNNImpute techniques. The domain knowledge of gene ontology is utilized for designing an imputation procedure^22^. The ontology tree is generated using the GO files, which are available on the GO official site. GO technique is a complement of the KNNImpute technique making improved imputation for small datasets. A knowledge-aided approach called Projection Onto Convex Sets (POCS) used the concept to organize all prior information into a related convex set. After that, using an iterative method to get a solution at the intersection of all such sets^23^. Stekhoven et al. proposed an iterative approach missforest using the concept of random forest^24^. Random forest a multiple imputation technique makes an average of numerous unpruned classification or regression trees. The built-in out-of-bag efficiency measuring method is used for calculating the performance of the random forest imputation technique. The technique performs better in the case when the dataset has complex interactions, non-linear relations, and high dimensionality. In the first phase, RF is trained on the available values, thereby imputing the missing values and this procedure is repeated. The technique takes more computation time as compared to KNNimpute but is lesser than MICE. Yoon et al. proposed Generative Adversial Imputation Nets (GAIN) based on the concept of generator and discriminator^25^. The generator’s objective is to impute the missing values by considering components of a real data vector, whereas the discriminator objective is to make a distinction between the available and imputed entries by considering the completed vector. The discriminator has the training principle of classification loss identifying the available and imputed value to be minimum, whereas the generator is trained in the manner in which the discriminator’s misclassification rate is maximized. The technique has the advantage of performing in the situation even if the whole dataset contains missing entries. One challenge for this technique is providing optimal supplement information called hints to the discriminator so that the resulting adversial process is the desired target. This additional imputation information confirms the generator to create samples as per the true data distribution. The limitation of knowledge-assisted approaches is that when the missing percentage increases, the performance of approaches becomes sub-optimal.

### Types of missing pattern

Missing patterns are classified into three types depending on the distribution of missingness.

1. Missing completely at random (MCAR)—In MCAR, there is no relation between data missing to any value of datasets missing or observed. In this mechanism, the missing data are an arbitrary subset of the dataset^26, 27^. This kind of missing pattern is usually not found in non-artificially produced datasets.
2. Missing at random (MAR)—The missing value is dependable on the available values. These available values can be used to estimate the missing values. MAR pattern of missingness is considered by most of the imputation procedures.
3. Missing Not at Random (MNAR)— In this category, the missing data can be analyzed using other instances of missing data, which makes it harder to predict the data. MNAR is also phrased as Not-Easily-Recoverable. This kind of missing pattern is negligibly used in any research. Mellenbergh et al. described these missing patterns and proposed methods to how these missing patterns can be avoided at the data collection phase^27^.

## Related work

The major part of this section focuses on graphs. This section describes the general definition of graphs, similarity graphs, the pairwise similarity between the data points, the specification of Laplacian, and a few characteristics.

### Similarity graph

For N data points representing genes, the similarity graph is defined as the matrix of the dimension $N\times N$. When the number of data points is more extensive, then there exists a limit on computer memory^28, 29^. Hence, there is a demand to make the matrix sparse, overcoming the drawback of storing the $N^2$ number of entries. For achieving this, there is a function in MATLAB known as Compressed Sparse Column (CSC). Different approaches to construct the similarity graph include the full similarity graph, K-nearest neighbor similarity graph, $\varepsilon$-neighborhood similarity graph.

#### Full similarity graph

In the full similarity graph, two vertices representing genes are connected whose similarity is not equal to 0. In other words, $v_i$ and $v_j$ are connected if $S_{ij}$ is not equal to 0. Gaussian similarity function is utilized to measure the distance between two data points.

2 $$\begin{aligned} S_{ij}=\exp \left( -\frac{d(g_i,g_j)^2}{2\sigma ^{2}}\right) \end{aligned}$$

The parameter $\sigma$ is utilized to control the size of the neighbors. Here d represents the distance measure, for example, the Euclidean measure. However, the above function does not provide the sparse structure that is desired.

#### K-Nearest neighbors similarity graph

The vertex $v_i$ representing gene can be connected to $v_j$ if the vertex $v_i$ is among the K nearest neighbors of $v_j$, where K is a specified number. This approach is better since it facilitates the matrix to be sparse. The basic illustration demonstrates that this always contributes to a directed graph. Two methods are used to convert into an undirected similarity graph: Normal K Nearest Neighbours and Mutual K Nearest Neighbours. For both categories, the similarity between two vertices is used for weighting the edges. In the former method, the vertices are connected if either of the vertices falls under another vertex’s nearest neighbor criterion. Whereas in the latter approach, the vertices are connected if one vertex is among the other’s closest neighbor and vice versa.

#### $\varepsilon$-neighborhood similarity graph

Two vertices representing genes are connected if the distance between them is lesser than a certain threshold whose value is greater than 0. There exists a numerically similar distance between the connected data points.

### Graph Laplacian

These are matrices used to represent the graph in a specific manner^30^. The properties of Graph Laplacian are described in this section so that the proposed methodology can use them. For an undirected graph G and weighted graph with adjacency matrix W and degree matrix D, the unnormalized graph laplacian is described as L=D-W. In comparison, the normalized graph laplacian is described as $L_{rw}=D^{-1}L$.

**Figure 2:**
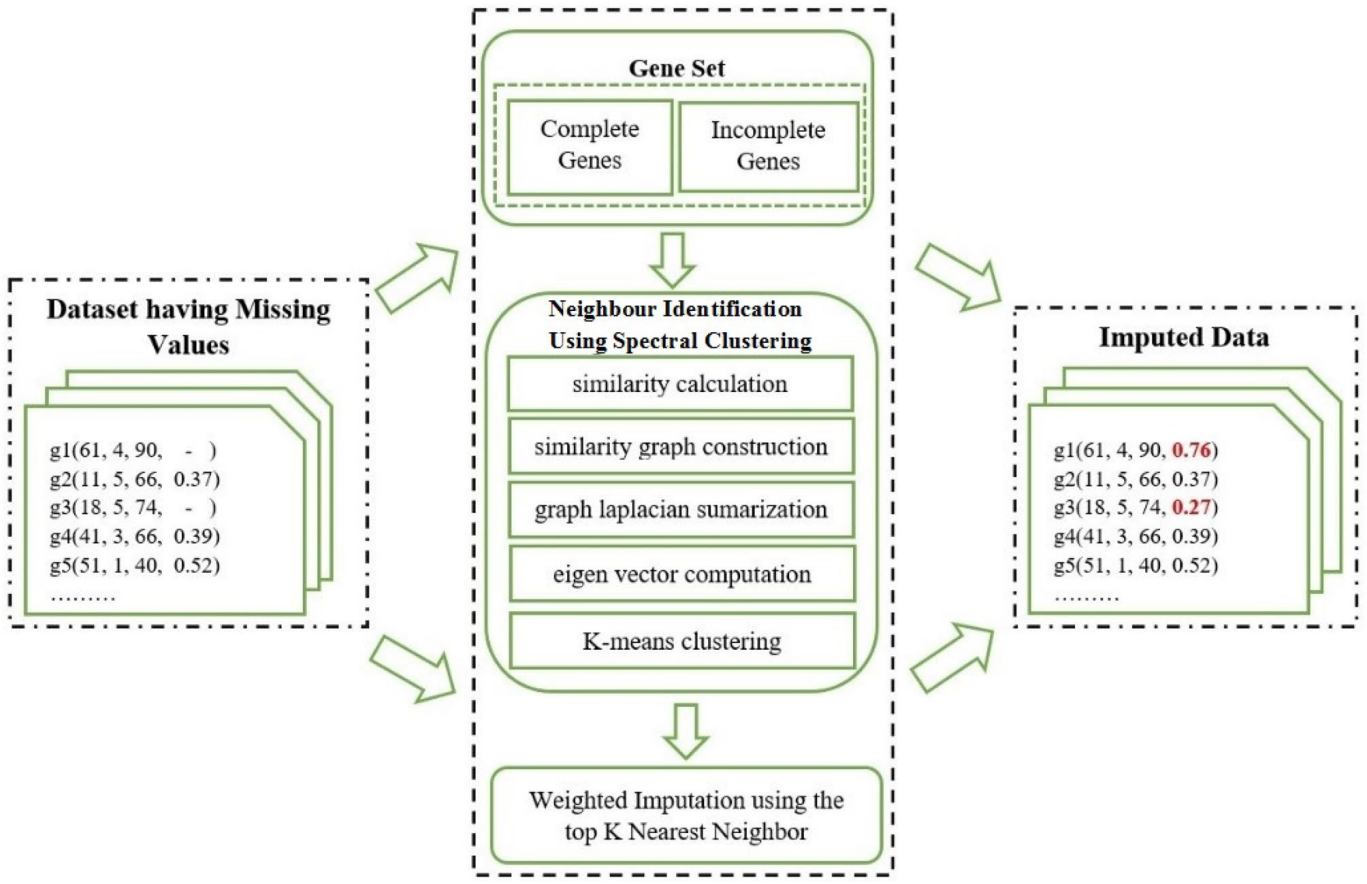
Block diagram of proposed method.

Figure 2 shows the imputation procedure into three steps. The entire process of the proposed technique is partitioned into three phases. In the first step, the complete gene set and incomplete gene set are identified from the full set of genes. In the second step, the neighbors of missing instances are determined based on their similarity. In the last step, the imputation is done using the previously discovered neighbors.

### Learning and evaluation

Schnabel et al. proposed a technique for dealing with selection biases, by adopting models and estimation approach from causal inference^31^. The technique is capable of handling the selection bias involved in the training and testing phase. Using unbiased performance calculators, an ERM structure is proposed that uses a matrix factorization procedure enabling learning recommendation structure responsible for selection bias with the property of scalability. The technique involves the missing structure of type MCAR and MNAR. In this paper, the MAR structured missing values are artificially inserted and thirty different datasets of a particular missing percentage are derived. The proposed algorithm and the existing algorithms are applied on these thirty datasets having a particular missing percentage. The average performance of a particular algorithm on these thirty datasets is declared is as the final performance of an algorithm for a particular missing percentage, which makes the learning and evaluation procedures to be unbiased.

## Proposed work

This paper presumed that missing data is MAR type, which means that missing data are deductible from other available data in some complicated way. The paper aims to estimate missing values using graph similarity combined with the K-means clustering algorithm. After that, the top KNN approach is combined with the weighted function criterion.

### Identification of complete and incomplete gene sets

A specific dataset having some of its data missings is classified into complete genes and incomplete genes. Incomplete genes are described as the rows having some of their missing column values. Similarly, the complete genes are identified as the rows of the dataset containing all the column values. The membership function describes a gene belonging to a specific cluster. Each gene belongs to a particular cluster with a specific membership degree. The absent value of an incomplete gene is determined utilizing the nearest neighbors of the missing instance. Each neighbor has a different closest distance to the missing instance; therefore, weighted concepts are used. In this paper, experiments reveal that the proposed local imputation procedure makes an accurate prediction compared to other procedures.

### Graph-based spectral clustering

One of the most popular data mining techniques is clustering which can be used to deal with missing data imputation. The clustering technique principle states that the genes are partitioned into a fixed number of clusters by identifying the similarity between genes. Minimizing the intra-cluster similarity and maximizing the inter-cluster similarity is the ultimate goal of clustering techniques. It is important to note that the distance and similarity existing between two gene instances are inverses of each other. In other words, in a cluster, the distance between two data points should be minimum, whereas the similarity between two data points should be high.

For analyzing the clusters from a dataset, there exist several algorithms. K-means and FCM are the most commonly used algorithms. These approaches tend to provide better clusters but have limitations. The approaches fail to cluster data points having a spherical shape. In comparison, the graph clustering technique (Spectral Clustering) can detect the correct patterns. For this, eigenvalues and eigenvectors are used in the technique. The spectral clustering technique is simple to implement, utilizing linear algebra concepts and provides accurate clusters for simple and typical types of datasets. 
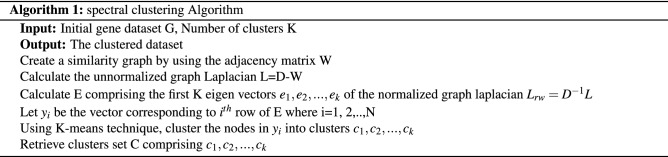


In algorithm 1, the K-means approach used a step in the spectral clustering approach consisting of a four-step procedure. Randomly selecting a fixed number of cluster centroid is the initial step. By identifying the closest centroid, each gene is assigned to a particular cluster. Cluster centroids are recalculated in the next step. Iteratively repetition of the process from the second step if the termination condition is not achieved.

The target gene neighbors containing missing sample values are identified as the genes falling in the same cluster. By utilizing these local cluster neighbors, imputation is made. Two types of approaches can be used for imputation after neighbor identification; the first is replacing the missing attribute with cluster centroid, secondly, replacing the missing attribute value with the nearest neighbor without the weighted function criterion. If the imputation is made by replacing the missing attribute value with the centroid attribute value, there is an imputation efficiency loss. Similarly, if neighbors are used without the weighted function criterion, efficiency loss occurs because as the number of neighbors is increased, the participation of irrelevant genes also increased. The weighted function criterion is used to overcome the drawback of the last two approaches, giving an efficient result even if there is a variation in the number of neighbors.

### Local similarity-based imputation

Assume that $R_j$ represents the instance having missing value for some attribute and $\{dist_1, dist_2,..., dist_{k'}\}$ represents the distances between the missing element to the available values $\{g_1, g_2, ..., g_{k'}\}$. Some steps for imputing the missing values are-

- *Step 1* For making the weighted imputation for the k’ nearest neighbors.
  3 $$\begin{aligned} P_i=\left( \frac{\frac{1}{dist_i}}{\sum _{i=1}^{k'} \frac{1}{dist_i}}\right) \end{aligned}$$
- *Step 2* For imputing each missing value at sample u of the type numeric $R_{ju}$ in $R_j$.
  4 $$\begin{aligned} I=\sum _{i=1}^{k'} P_i g_{iu} \end{aligned}$$
  Here $g_{iu}$ represents the correspondent value of $g_i$.
- *Step 3* For imputing the missing value of the type categorical, the expected value of the corresponding attribute value is calculated as
  5 $$\begin{aligned} E_{u_v}=\sum _{i=1}^{k''} P_i \end{aligned}$$
  where k” represents the number of available values $u_v$ for the attribute u, the value having the highest expected value is utilized for making imputation of missing $R_{ju}$.

Algorithm 2 describes the complete procedure of imputation. At the initial stage, the dataset is clustered using spectral clustering. At the next step, the instances containing the missing entries are determined based on their top KNN utilizing hybrid weighted distances. 
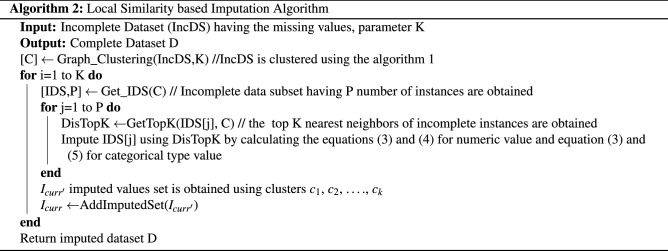


## Experiments and analysis

### Dataset description

Table 2 summarizes the gene expression dataset used in the research. The second column represents the total number of genes and the total number of samples in the original dataset. For testing the accuracy of the procedure, only available genes are considered for research. The third column represents the number of genes that are complete that is not having an absent value. The fourth column explains the missing rate. It is deducible that the dataset comprises a differing degree of missing data. All microarray datasets used in this paper are accessible online.

**Table 2:** Summary of the datasets.

Datasets	Original matrix (${g\times s}$)	Complete matrix (${g\times s}$)	Missing rate (%)	References
GDS1761	$9706\times 64$	$8849\times 64$	0.15	^32^
GDS5232	$32878\times 50$	$16819\times 50$	48.05	^33^
GDS2735	$22575\times 46$	$20844\times 46$	7.66	^34^
GDS1210	$7129\times 30$	$7122\times 30$	0.02	^35^

The GDS1761 dataset contains the examination of cell lines from tumors in different tissues and organs. These cell lines are commonly used as neoplastic research models. The outcome of the analysis can identify gene-expressed variations in the cell lines and Vivo tumor interactions. GDS1761 consists of a missing percentage of 0.15% since 857 out of 9706 genes consist of at least one missing value.

GDS5232 consists of the experimental data of primary colorectal cancer tumors gathered from different aged patients. The patients’ age varies from a young age of 28 to 53 years to old-aged patients 69 to 87 years. The collected data is used for the identification of the earliest colorectal cancer-related genes. GDS5232 consists of a missing percentage of 48.05% since 16059 out of 32878 genes consist of at least one missing value.

GDS2735 consists of experimental data related to sorted peripheral blood lymphocytes collected from the patients suffering from melanoma. These subunits have involvement in antitumor responses and are adversely affected by cancer. The outcome gives a perception of the immune dysfunctionality process in cancer. GDS2735 consists of a missing percentage of 7.66% since 1731 out of 22575 genes consist of at least one missing value.

GDS1210 contains 22 primitive specialized gastric tissue expression characterization. Entire gastric cancer tissues were studied with metastasis and histology. The outcome gives the perception of gastric cancer growth and variety. GDS1210 consists of a missing percentage of 0.02% since 7 out of 7129 genes consist of at least one missing value.

**Figure 3:**
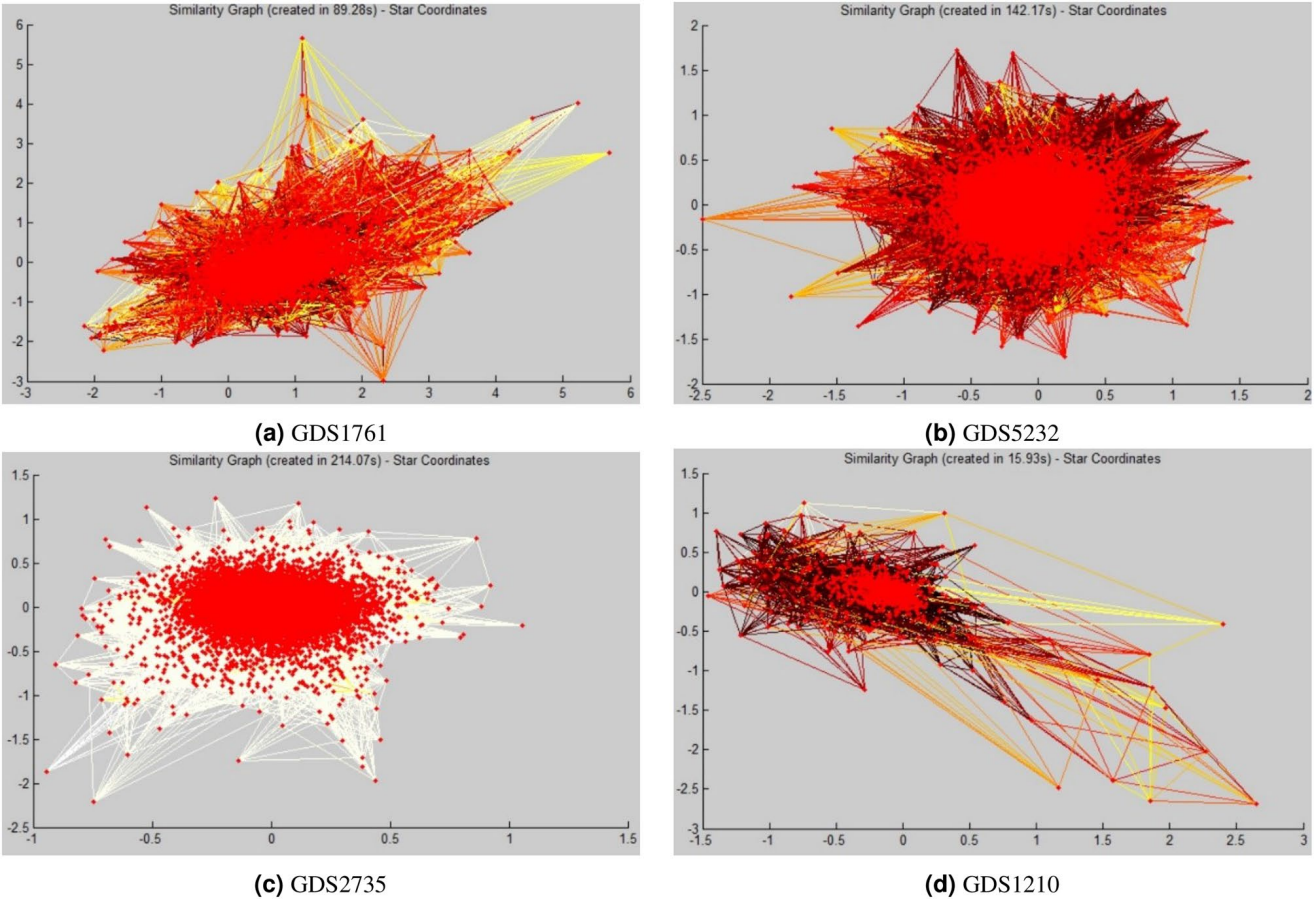
Similarity graph for four gene expressed dataset (**a**) GDS1761 (**b**) GDS5232 (**c**) GDS2735 (**d**) GDS1210.

**Figure 4:**
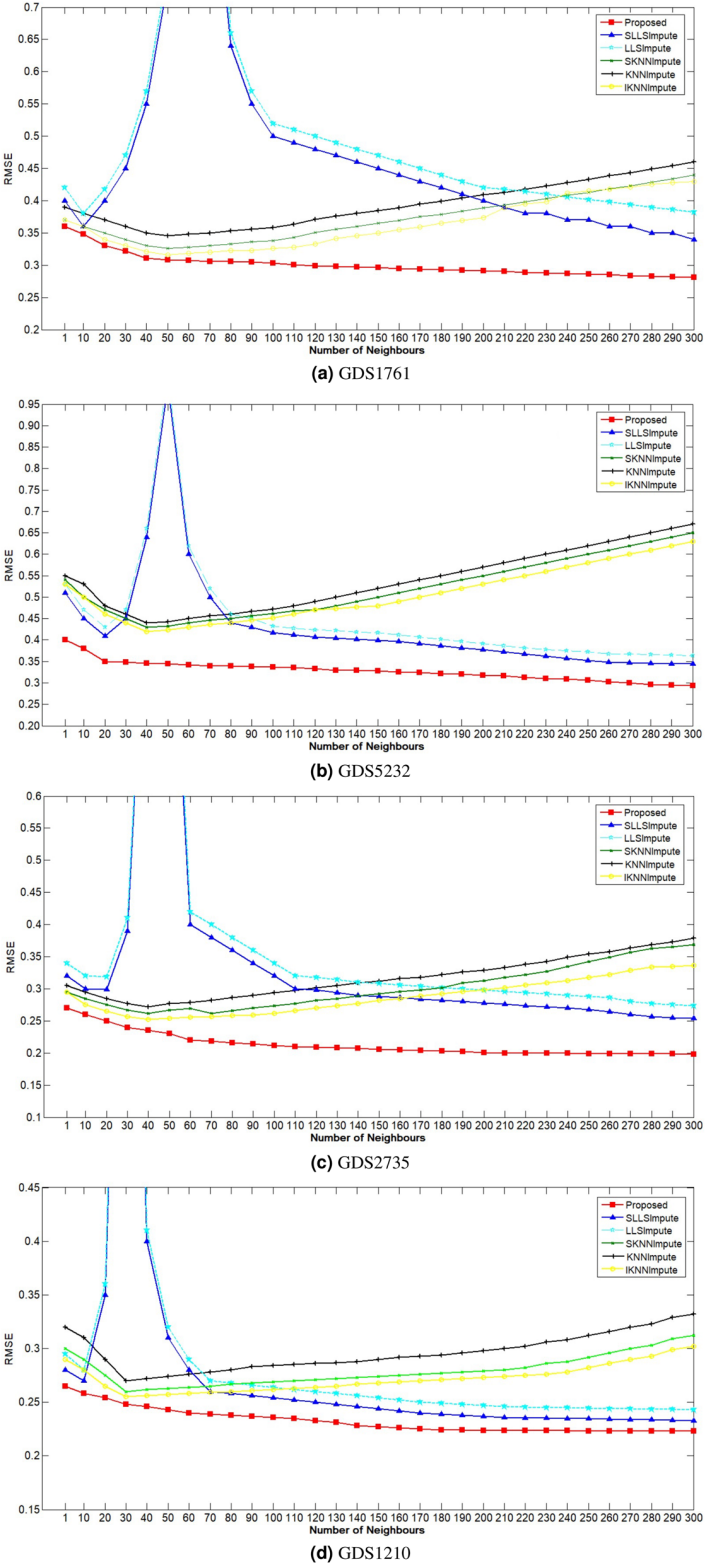
RMSE vs. the number of neighbors on the four gene expressed dataset (**a**) GDS1761 (**b**) GDS5232 (**c**) GDS2735 (**d**) GDS1210.

### Experimental design

Extensive experiments are performed on four gene expression datasets to illustrate the capability of the proposed procedure. For generalizing the behavior of proposed techniques, experiments with more datasets having the varying number of rows and columns, different missing pattern and different missing percentage is required. For experimenting, some portion of the data is deleted so that the dataset contains 5%, 10%, 15%, 20%, 25% of missing percentage. Figure 3 shows the similarity graph of all four genes expressed dataset. Each vertex represents a gene, and the edge between them represents the similarity between them. The data points shown to be far from the others (dense cluster) are treated as an outlier. These similarity graphs are used as input to the spectral clustering algorithm. The spectral clustering algorithm makes it easier to identify the number of neigbors for a particular gene.

**Figure 5:**
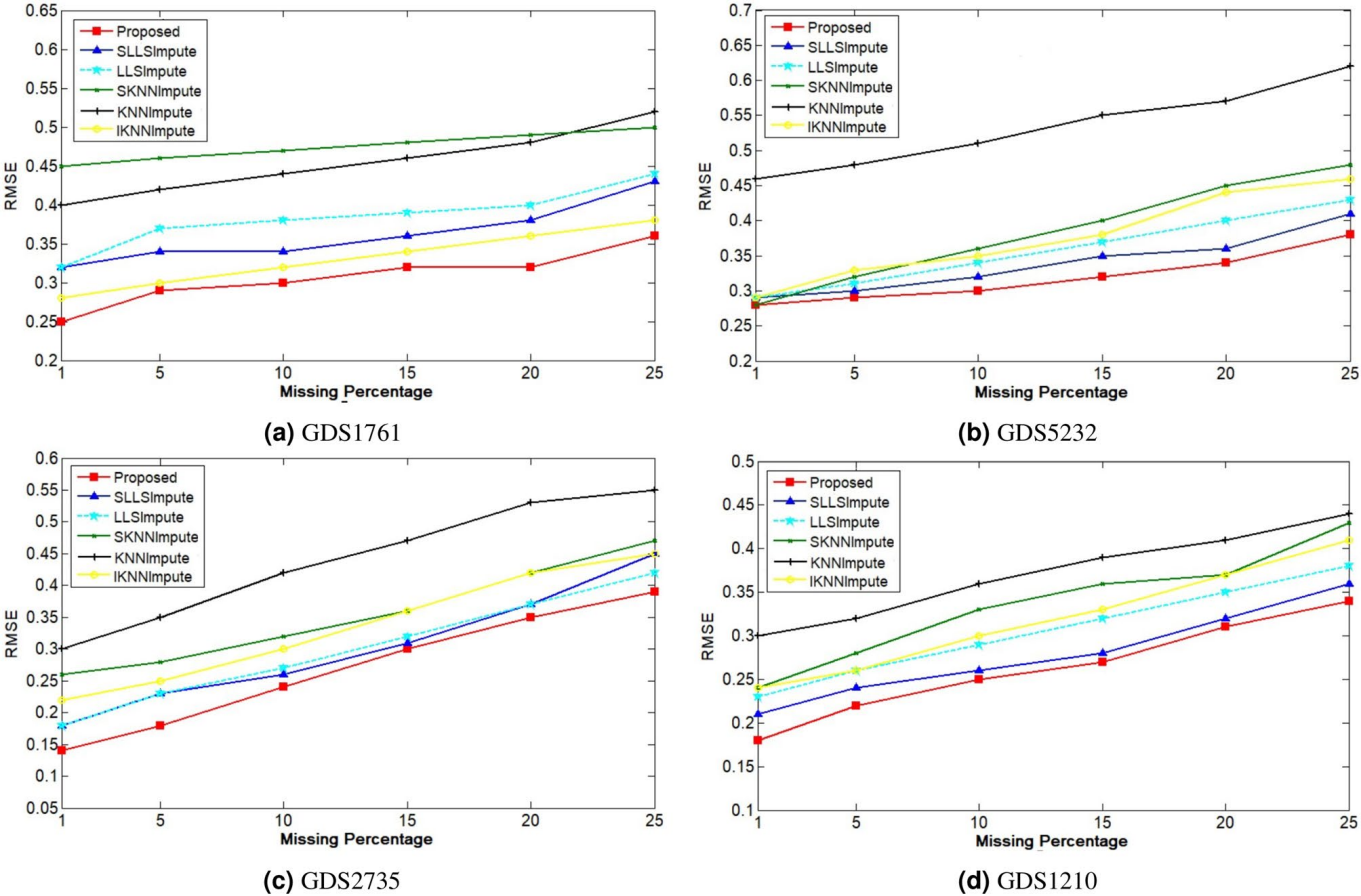
RMSE comparison of proposed technique with other techniques having different missing percentage on the four gene expressed dataset (**a**) GDS1761 (**b**) GDS5232 (**c**) GDS2735 (**d**) GDS1210.

### Selection of parameter

Selecting the optimum number of neighbors is crucial for local imputation methods, including KNNImpute, IKNNImpute, SKNNImpute, SLLSImpute, LLSImpute, and the proposed technique. Experiments are performed to know the optimal value of the number of neighbors for each technique by investigating the relation between the number of neighbors and the RMSE for a fixed missing percentage of 5%. The parameter value ranging from 1 to 300 experimented thirty times. Figure 4 represents the average RMSE of the six techniques. The X-axis shows the number of neighbors, whereas the Y-axis shows the RMSE value. For SLLSImpute and LLSImpute, as the value of the number of neighbors is approaching the number of samples, the RMSE is rising quickly, and then RMSE is degrading with the increment of the number of neighbors. Whereas for KNNImpute, IKNNImpute, and SKNNImpute, the RMSE firstly degrades using the incremented neighbors and then increases with the increasing value of neighbors. The reason behind it is that as the number of neighbors increases, the participation of relevant and irrelevant genes also increased, causing the prediction accuracy to be decreased. The proposed technique’s performance can not be worst even if the number of neighbors is kept high since the weighted function criterion does not allow the irrelevant gene to be considered in the imputation process.

### Performance evaluation

Figure 5a–d shows the comparison of imputation error by various local imputation procedures. Before applying the imputation technique, thirty distinct sets having the same missing percentages are made; all imputation techniques are implemented on these sets; after that, the average of those thirty RMSE is computed. Lesser RMSE value shows that the prediction is made accurately. It is a challenge for each imputation technique to perform well even when the missing percentage rises to 25%. It can be derived from the figures that all imputation techniques have approximately similar performance when the missing percentage is low. But, a rise in the missing percentage makes the performance of each imputation technique to be noticeable. For comparing the proposed algorithm’s performance, KNNImpute, SKNNImpute, IKNNImpute, SLLSImpute, and LLSImpute are utilized. The proposed technique makes accurate predictions than other imputation procedures, even if the missing percentage rises to 25%. It is observed that the SLLSImpute technique achieves better imputation accuracy as compare to the KNN derived techniques, which shows that regression models are better to the nearest neighboring technique. The hyper-parameters required for designing an imputation algorithm are set in such a manner that the procedure gains the best efficiency. For example, by looking at Fig. 4 having 5% missingness, it can be easily derived that the SKNNImpute, KNNImpute, and IKNNImpute achieve better results when the number of neighbors is equivalent to the number of samples in the dataset. Similarly, all the imputation procedures used for comparing the performance against the proposed algorithm have their optimal parameter values. Experimental results show that the proposed technique has a lower RMSE value even if the missing percentage is kept high compared to other local imputation techniques.

## Conclusion and future work

In this paper, the local imputation method utilizing the clustering principle has been applied to the microarray gene-expressed dataset. However, graph theory usage for similarity graphs, including using the matrix for clustering, imposes a higher complexity, making the complete procedure cumbersome. For reducing the computational cost, some techniques to make the matrix sparse that is using the K nearest neighbor or $\varepsilon$-neighborhood for similarity graph construction. Research results on gene data reveal that the proposed method outperforms other current imputation methods. Exact imputation results can be achieved by the selection of correct parameters and operational framework. Finally, the proposed technique can be summarized as a precise estimation technique of missing values with improved efficiency.

Since the distribution of missing data is crucial for evaluating an imputation procedure, future work requires investigation of the relationship between an imputation procedure’s accuracy and a specific missing data pattern. Secondly, the proposed technique may also be implemented on other application datasets that also suffer from missing data, such as proteomics and RNA sequence datasets. RNA sequence data collected from highly parallel sequenced techniques could contain missing read counts. These datasets contain similar expression patterns and reveal local similarity constructs within a cell population, resembling that similarity between genes or cells may be used to impute the missing entries. Besides utilizing microarray data intensity, read the RNA sequence data utilize counts for measuring the gene expression, explicitly predicting the missing value-the countability feature of RNA sequence data required to be considered. Thus, the proposed technique can be implemented on RNA sequence data, and then testing its accuracy is another important future work.

## Data Availability

All datasets used in this paper are accessible online https://www.ncbi.nlm.nih.gov/sites/GDSbrowser/.
